# Quantitative Assessment of Deep Gray Matter Susceptibility and Correlation With Cognition in Patients With Liver Cirrhosis

**DOI:** 10.1002/brb3.70240

**Published:** 2025-01-08

**Authors:** Wenjun Wu, Yu Su, Ziji Qin, Jiamin Kang, Dongqiao Xiang, Dingxi Liu, Chuansheng Zheng, E. Mark Haacke, Lixia Wang

**Affiliations:** ^1^ Department of Radiology, Union Hospital, Tongji Medical College Huazhong University of Science and Technology Wuhan China; ^2^ Hubei Province Key Laboratory of Molecular Imaging Wuhan China; ^3^ Department of Radiology The People's Hospital of Guangxi Zhuang Autonomous Region Guangxi Academy of Medical Sciences Nanning China; ^4^ Department of Radiology, Wuhan No. 1 Hospital, Tongji Medical College Huazhong University of Science and Technology Wuhan China; ^5^ Magnetic Resonance Innovations Bingham Farms Michigan USA; ^6^ Department of Radiology Wayne State University Detroit Michigan USA

**Keywords:** liver cirrhosis, deep gray matter, quantitative susceptibility mapping, cognitive impairment, metal accumulation

## Abstract

**Background and Objectives:**

Accumulation of metals quantified by quantitative susceptibility mapping (QSM) in deep gray matter (DGM) and their impact on cognition have not been studied in patients with liver cirrhosis. This study aims to use QSM to investigate the association between DGM susceptibility and cognition in cirrhotic patients.

**Methods:**

Thirty cirrhotic patients and 30 age‐, gender‐, and education‐matched controls were imaged using a multiecho gradient‐echo sequence for QSM analysis in a 3T scanner. The susceptibility values were determined for the caudate nucleus (CN), putamen (PU), globus pallidus (GP), thalamus (TH), red nucleus (RN), substantia nigra (SN), and dentate nucleus (DN). All subjects underwent number connection test A (NCT‐A), digit substitution test (DST), and Montreal Cognitive Assessment (MoCA). Comparisons between the two groups and the correlation between the susceptibility values and neuropsychological scores were analyzed.

**Results:**

The susceptibility values of bilateral CN, TH, and RN were significantly lower in cirrhotic patients. Cirrhotic patients exhibited significantly prolonged NCT‐A time and decreased DST and MoCA scores. The NCT‐A, DST, MoCA, and sub‐domain scores were correlated with susceptibility values of RN, DN, SN, and CN, respectively. The susceptibility value of the left RN was a predictor variable for the DST, MoCA, and visuospatial–executive scores; those of the right CN and left RN were predictor variables for the naming score, and that of the left SN was an independent predictor variable for the language score.

**Conclusions:**

Altered susceptibility values of DGM measured by QSM are potential quantitative indicators of cognitive impairment in cirrhotic patients.

## Graphical abstract

The impact of brain metal deposition on cognitive decline in patients with liver cirrhosis remains unclear. Quantitative susceptibility mapping (QSM) was employed to assess deep gray matter, and its association with cognition was examined. Our findings demonstrate a significant correlation between the susceptibility of the red nucleus, substantia nigra, and caudate nucleus and cognitive function in individuals with liver cirrhosis.

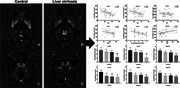

## 1 Introduction

Liver cirrhosis causes subclinical cognitive dysfunction known as minimal hepatic encephalopathy (MHE) (Weissenborn and Butterworth 2019; Bajaj et al. 2020). Though without identifiable clinical manifestations, MHE still negatively affects quality of life, predisposes to clinical hepatic encephalopathy, and reduces lifespan (Bajaj et al. 2020; Montagnese and Bajaj 2019). Chronic liver failure induces multiple pathophysiological alterations affecting the brain (Butterworth 2019; Felipo 2013). Recently, magnetic resonance spectroscopy (MRS) (Zeng et al. 2020), functional magnetic resonance imaging (fMRI) (Ye et al. 2020), arterial spin labeling (ASL) (Li et al. 2017), diffusion tensor imaging (DTI)/diffusion kurtosis imaging (DKI) (Sun et al. 2020), and high‐resolution structural MRI (Chen et al. 2020) have been applied to reveal changes in brain metabolism, function, and structure in cirrhotic patients. However, current studies on the changes in cirrhosis‐related brain metal content are still limited, which may provide new insights into the underlying mechanism of MHE.

Quantitative susceptibility mapping (QSM) is a reliable method to measure susceptibility changes induced by metal, especially iron, in the brain (Haacke et al. 2020; Vinayagamani et al. 2021; Liu et al. 2012; Zheng et al. 2013). Iron deposition in the deep gray matter (DGM) plays an important role in the pathophysiological process of several neurodegenerative diseases, such as Alzheimer's disease (AD) and Parkinson's disease (PD) (Rouault 2013; Uchida et al. 2022). A few previous studies have used T2* MRI or QSM to suggest abnormal DGM iron deposition in cirrhotic patients, but the results are inconsistent (Xia et al. 2015; Liu et al. 2013). Recently, another QSM study showed significantly lower susceptibility values in the globus pallidus and dental nucleus in patients with pallidal T1 hyperintensity (Lee et al. 2018). In a postmortem study, excessive manganese and copper accumulation were found in the basal ganglia of patients with chronic liver failure (Klos et al. 2006). Although iron is a major factor in altering regional magnetic susceptibility due to its significantly higher abundance in the brain, manganese and copper deposits should not be overlooked in certain diseases (Lee et al. 2018, Li et al. 2020). Dysregulation of metal, such as iron, balance may interfere with key enzymatic activities, thereby inducing oxidative stress and mitochondrial dysfunction, thereby altering cognitive status (Chen et al. 2019). However, altered regional susceptibility of DGM in cirrhotic patients and its relationship with cognitive status have not been fully studied.

Conventional MRI demonstrates some characteristic signal abnormality such as T1 hyperintensity in chronic liver failure and T2 hyperintensity in end‐stage liver failure. Although the sources of these changes likely have different origins, both share similar distribution patterns involving DGM (Krieger et al. 1995; Kim et al. 2008). We hypothesize that metal‐induced altered susceptibility of DGM is associated with cognitive function in cirrhotic patients. This study used QSM to investigate the altered susceptibility of DGM and its association with cognitive impairment in patients with liver cirrhosis.

## 2 Materials and Methods

### 2.1 Subjects

This cross‐sectional study was approved by the Institutional Ethics Committee (No. UHCT21810). All subjects were informed and signed written consent before participation. Thirty cirrhotic patients (21 men, 9 women; age range, 31–71 years; mean age, 53.9 years) were recruited from our hospital between October 2020 and June 2021. Cirrhosis was diagnosed according to clinical signs, laboratory examination, and abdominal computed tomography (CT) or MRI findings. The inclusion criteria included: the patients had (i) laboratory indicators of cirrhosis and typical abdominal CT or MRI findings of cirrhosis; (ii) ability to complete the neuropsychological assessments; (iii) no other neurological and psychiatric disorders, drug abuse, head trauma, neoplasm, and operation according to their medical history. The exclusion criteria included: (i) the patients had severe claustrophobia or other MRI contraindications; (ii) the quality of brain MR images is not sufficient for post‐processing and evaluation due to motion or other artifacts; (iii) conventional brain MR images showed obvious structural abnormalities. Thirty healthy subjects matched for age, sex, and education were included in the study as a control group (17 men, 13 women; age range, 30–73 years; mean age, 51.8 years) through advertisements. All subjects underwent neuropsychological assessments before MRI scanning.

### 2.2 Neuropsychological Assessments

The number connection test A (NCT‐A) was used to measure the cognitive–motor abilities of subjects by recording the time needed to connect numbers from 1 to 25. NCT‐A completion time in seconds was recorded. The digit substitution test (DST) was used to measure information processing speed and attention switching by replacing randomized letters with an appropriate digit indicated by the key. The DST score was the number of correct substitutions made in 60 s. An NCT‐A time > 2 standard deviation (SD) or a DST score > 2 SD from that of controls was considered abnormal. The Montreal Cognitive Assessment (MoCA) scale was used to evaluate a subject's overall cognitive status. It has 30 brief questions assessing seven aspects of cognitive sub‐domains including visuospatial/executive function, naming, attention, abstraction, language, delayed memory, and orientation on a scale of 0–30 (the higher the score, the better the function).

### 2.3 Magnetic Resonance Imaging

MRI was performed using a 3.0 T MR scanner (MAGNETOM Skyra, Siemens Healthcare, Erlangen, Germany) with a 32‐channel head coil. Routine axial T1WI, FLAIR, DWI, sagittal T2WI, and strategically acquired gradient echo (STAGE) (Chen et al. 2018) sequences were performed. STAGE consisted of two double‐echo (DE) GRE scans employing a pair of optimal flip angles: (1) low flip angle (FA) axial DE PDW: TR/TE = 7.5/17.5 ms, field‐of‐view (FOV) = 256 mm × 256 mm, FA = 6°, matrix = 384 × 144, slice thickness (TH) = 2 mm, number of slices = 64, voxel size = 0.67 × 1.33 × 2.0 mm^3^ (interpolated to 0.67 × 0.67 × 2.0mm^3^); (2) high FA axial DE T1W: TR/TE = 8.75/18.75 ms, FOV = 256 mm × 256 mm, FA = 24°, matrix = 384×144, TH = 2 mm, number of slices = 64, and a voxel size = 0.67 × 1.33 × 2.0 mm^3^ (interpolated to 0.67 × 0.67 × 2.0 mm^3^). The acquisition time of STAGE was 5 min 36 s.

### 2.4 Image Processing

QSM was performed using STAGE processing software (STAGE version 2.1.4) with the following steps: skull removal using brain extraction tool (BET), phase unwrapping, background field removal using sophisticated harmonic artifact reduction for phase data (SHARP) algorithm, and an inverse filter with a k‐space threshold of 0.1. More details on the methodology can be found in previous articles on STAGE (Haacke et al. 2020; Chen et al. 2018; Wang et al. 2018).

**FIGURE 1:**
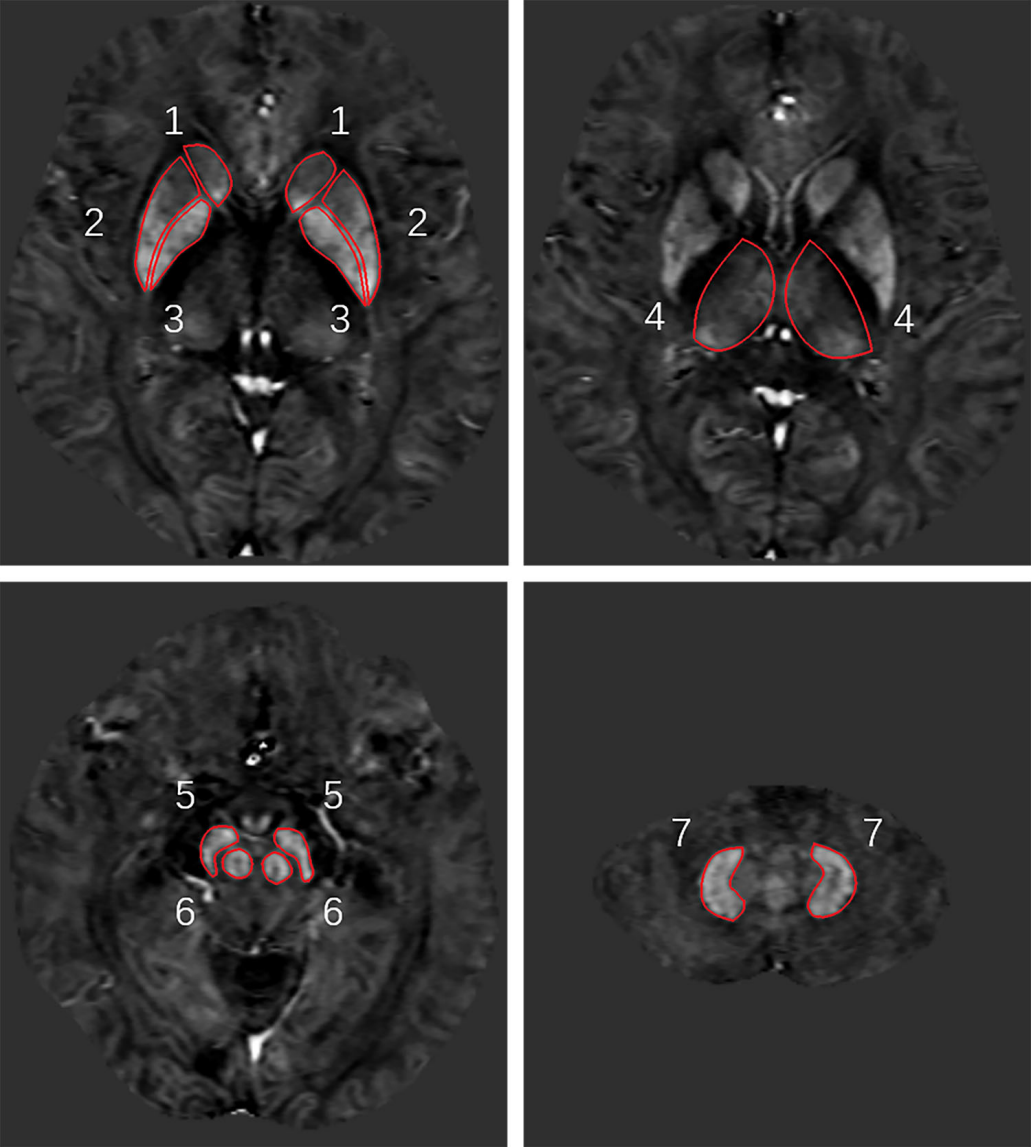
Regions of interest (ROI) demarcated on the quantitative susceptibility mapping (QSM) images. 1 = caudate nucleus (CN); 2 = putamen (PU); 3 = globus pallidus (GP); 4 = thalamus (TH); 5 = substantia nigra (SN); 6 = red nucleus (RN); 7 = dentate nucleus (DN).

Two trained neuroradiologists demarcated the regions of interest (ROIs) based on the anatomy on the STAGE maps and recorded the susceptibility values. The ROIs included bilateral caudate nucleus (CN), putamen (PU), globus pallidus (GP), thalamus (TH), red nucleus (RN), substantia nigra (SN), and dentate nucleus (DN). The ROIs of the DGM were demarcated along the contour of each structure (Figure 1). The final susceptibility values were determined as the average of measurements by the two neuroradiologists.

### 2.5 Statistical Analysis

Statistical analyses were performed using SPSS software (version 26.0; SPSS, Inc., Chicago, IL, USA), and results were expressed as mean ± SD. Comparisons of the age, education, neuropsychological scores, and susceptibility values were performed using an independent two‐sample *t*‐test, with the significance level (α) adjusted by the Bonferroni correction. A χ2 test was used to compare the proportion of gender. Pearson correlation analysis was used for the relationship between susceptibility values and Child–Pugh score, as well as blood ammonia levels. Partial correlation analysis was used to evaluate the relationship between the susceptibility values and neuropsychological scores in patients with liver cirrhosis. Age and education were used as covariates. The correlation between blood ammonia levels and neuropsychological scores was analyzed using both Pearson correlation and partial correlation analysis. The MoCA sub‐domain analysis also compares susceptibility values across different scores using an independent two‐sample *t*‐test. Stepwise multiple linear regression analysis was performed using neuropsychological scores as dependent variables and age, education, and susceptibility values of different DGM as independent variables. A *p* value less than 0.05 was considered to indicate statistical significance.

## 3 Results

There were no significant differences in age (*p* = 0.488), gender (*p* = 0.292), and education level (*p* = 0.979) between the two groups. The average Child–Pugh score of cirrhotic patients was 6.3, and the Child–Pugh classification included 19A/10B/1C. Compared with controls, cirrhotic patients had higher NCT‐A scores (*p* = 0.029), lower DST scores (*p* = 0.002), lower MoCA total scores (*p* = 0.001), lower visuospatial/executive function scores (*p* = 0.001), lower naming scores (*p* = 0.001), lower attention scores (*p* = 0.001), lower language scores (*p* = 0.001), lower delayed memory scores (*p* = 0.001), lower orientation score (*p* = 0.030), and no significant difference in abstraction scores (*p* = 0.763; Table 1).

**TABLE 1:** Demographic, clinical data and neuropsychological scores of all subjects.

	Normal controls(*n* = 30)	Cirrhotic patients(*n* = 30)	*p* value
Age(years)^b^	51.77 ± 10.51	53.90 ± 12.6	0.488
Gender(male/female)^a^	17/13	21/9	0.292
Education(years)^b^	10.27 ± 3.56	10.24 ± 3.70	0.979
Child–Pugh scores (score)	—	6.3 ± 1.62	—
Child–Pugh class	—	19A/10B/1C	—
Blood ammonia level (µmol/L), *n* = 11	—	35.45 ± 22.92	
Pallidal T1 hyperintensity (*n*)	—	5	—
Causes of liver cirrhosis		Hepatitis B (*n* = 22) Biliary diseases (*n* = 5) Autoimmune hepatitis (*n* = 2) Hemochromatosis (*n* = 1)	
NCT‐A (s)^b^	48.37 ± 20.17	65.37 ± 36.49	0.029^*^
DST (score)^b^	38.43 ± 14.33	28.07 ± 9.63	0.002^*^
MoCA score^b^	26.97 ± 2.30	20.59 ± 5.96	0.001^*^
Visuospatial‐executive score^b^	4.47 ± 0.73	3.17 ± 1.26	0.001^*^
Naming score^b^	2.83 ± 0.46	2.07 ± 0.884	0.001^*^
Attention score^b^	5.43 ± 0.77	4.07 ± 1.25	0.001^*^
Language score^b^	2.40 ± 0.62	1.34 ± 1.01	0.001^*^
Abstraction score^b^	1.40 ± 0.62	1.34 ± 0.77	0.763
Delayed recall score^b^	3.50 ± 1.00	2.24 ± 1.77	0.001^*^
Orientation score^b^	6.00 ± 0.00	5.59 ± 1.02	0.030^*^

^a^ The *p* value was obtained using the χ2 test.

^b^ The *p* value was obtained using the independent two‐sample *t*‐test.

* *p* < 0.05.

**FIGURE 2:**
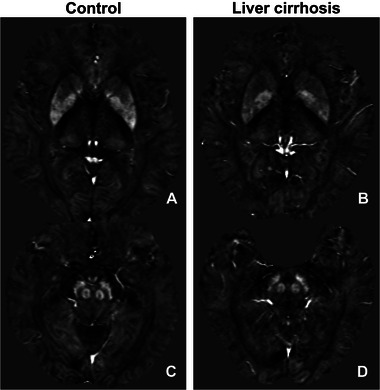
Representative QSM images of control (a,c) and liver cirrhosis (b,d). Liver cirrhosis exhibits a relatively lower magnetic susceptibility in basal ganglia and midbrain.

**FIGURE 3:**
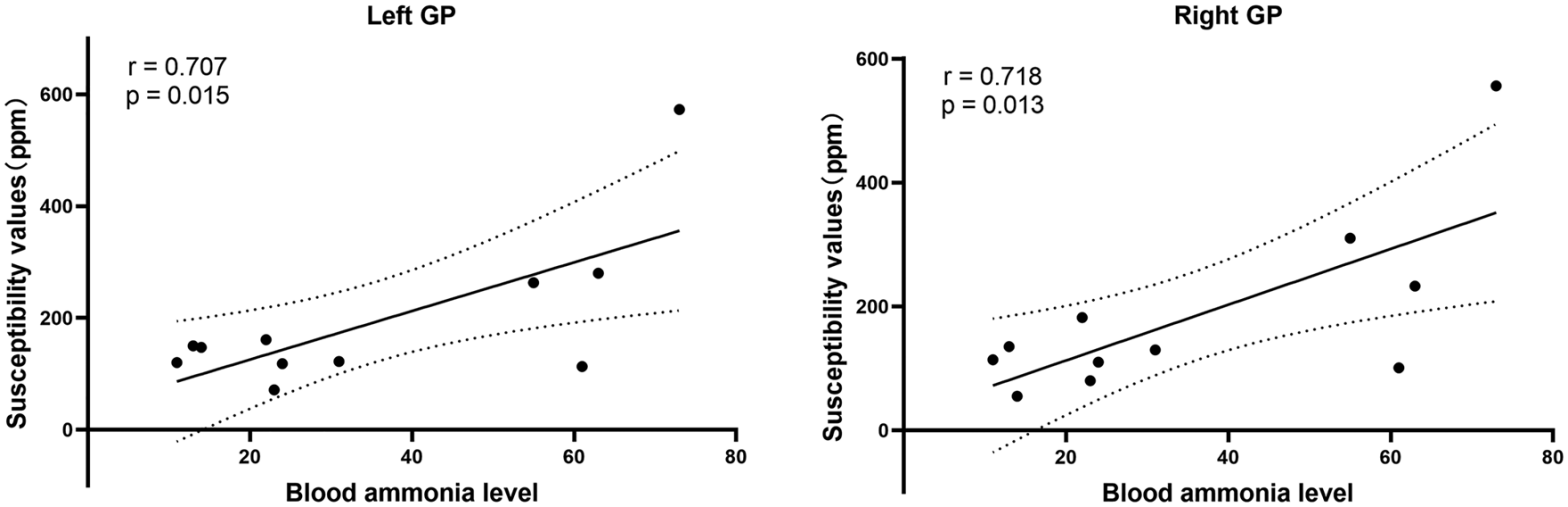
Correlation between blood ammonia level and susceptibility values in globus pallidus.

Among the controls, the average susceptibility values of CN, PU, GP, TH, RN, and SN were 41.2, 46.2, 140.4, −0.7, 129.4, and 145 ppb (parts per billion), respectively, which were significantly correlated with the postmortem brain iron concentrations in the corresponding regions reported by Hallgren and Sourander (*r* = 0.974, *p* < 0.001). Compared with controls, cirrhotic patients had significantly decreased susceptibility values in the bilateral CN, TH, and RN (all *p* < 0.05; Table 2 and Figure 2). The Child–Pugh score was positively correlated with right GP (*r* = 0.393, *p* = 0.039) and negatively correlated with right and left PU (*r* = −0.429, *p* = 0.023; *r* = ‐0.412, *p* = 0.029). The blood ammonia level was positively correlated with left and right GP (*r* = 0.707, *p* = 0.015; *r* = 0.718, *p* = 0.013; Figure 3). No significant correlation was found between blood ammonia levels and neuropsychological scores (all *p* > 0.05).

**TABLE 2:** Susceptibility values [ppb (×10−9)] differences between the groups.

ROI	Normal controls (*n* = 30)	Cirrhotic patients (*n* = 30)	*p* value	Effect size^a^
Right CN	40.66 ± 13.35	32.82 ± 13.01	0.025^*^	0.59
Left CN	41.81 ± 11.18	34.30 ± 12.52	0.018^*^	0.63
Right GP	141.33 ± 33.62	157.14 ± 103.85	0.433	0.20
Left GP	139.38 ± 31.00	165.65 ± 99.08	0.175	0.36
Right PU	46.51 ± 12.47	50.65 ± 33.08	0.525	0.17
Left PU	45.83 ± 15.19	55.29 ± 31.88	0.149	0.38
Right TH	−0.58 ± 8.91	−10.69 ± 10.74	0.001^*^	1.02
Left TH	−0.79 ± 8.27	−11.74 ± 11.94	0.001^*^	1.07
Right RN	129.68 ± 37.04	102.14 ± 36.23	0.005^*^	0.75
Left RN	129.03 ± 38.63	100.29 ± 34.63	0.004^*^	0.78
Right SN	142.93 ± 31.13	129.98 ± 38.23	0.158	0.37
Left SN	147.01 ± 30.90	128.21 ± 45.59	0.067	0.48
Right DN	111.25 ± 37.44	106.17 ± 38.19	0.605	0.13
Left DN	115.04 ± 35.16	110.06 ± 42.34	0.622	0.13

^a^ Cohen's d value.

All *p* values were obtained using the independent two‐sample *t*‐test.

* *p* < 0.05.

**FIGURE 4:**
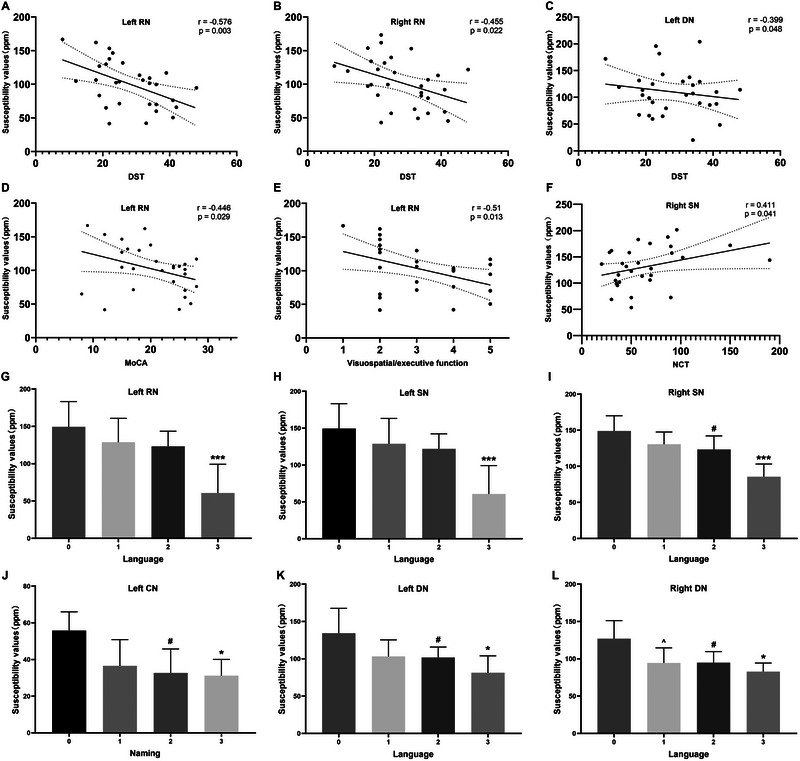
(a–f) Correlations between the susceptibility values of different DGM and the neuropsychological scores in the patients with liver cirrhosis. (g–l) MoCA sub‐domain analysis compares susceptibility values across different scores. *** = *p* < 0.05 between score 3 and scores 0, 1, 2; * = *p* < 0.05 between score 3 and score 0; **^#^** = *p* < 0.05 between score 2 and score 0; **^** = *p* < 0.05 between score 1 and score 0.

In patients with liver cirrhosis, the MoCA total scores, visuospatial/executive function scores, language scores, and DST scores were negatively correlated with the susceptibility values of the left RN (*r* = −0.446, *p* = 0.029; *r* = −0.51, *p* = 0.013; *r* = −0.443, *p* = 0.034; *r* = −0.576, *p* = 0.003, respectively), and the DST scores were negatively correlated with the susceptibility values of the right RN (*r* = −0.455, *p* = 0.022). The DST scores were negatively correlated with the susceptibility values of the left DN (*r* = −0.399, *p* = 0.048), and the language scores were negatively correlated with left and right DN (*r* = −0.567, *p* = 0.005; *r* = −0.575, *p* = 0.004, respectively). The naming scores were negatively correlated with the susceptibility values of the right CN (*r* = −0.479, *p* = 0.021). The NCT‐A scores were positively correlated with the susceptibility values of the right SN (*r* = 0.411, *p* = 0.041), and the language scores were negatively correlated with the susceptibility values of the left and right SN (*r* = −0.494, *p* = 0.017; *r* = −0.51, *p* = 0.013). The MoCA sub‐domain analysis also revealed a decrease in susceptibility values as the neuropsychological scores increased (Figure 4a–l).

In patients with liver cirrhosis, the multivariate analysis showed that the susceptibility values of the left RN exerted significant effect on the DST, MoCA, and visuospatial‐executive function scores (β = −0.383, *p* = 0.003; β = −0.360, *p* = 0.029; β = −0.375, *p* = 0.013), and the susceptibility values of the right CN and left RN exerted significant effect on the naming scores (β = −0.391, *p* = 0.035; β = −0.382, *p* = 0.039), and the susceptibility values of the left SN exerted significant effect on the language scores (β = −0.491, *p* = 0.013). Only the predictor variables with statistically significant dependence were listed (Table 3).

**TABLE 3:** Multiple linear regression analysis between specific neuropsychological performance and age, education, and susceptibility values of DGMs in cirrhotic patients.

	Factors	B	Standardized B (β)	*T*	*p*	*R* of model	*R* ^2^ of model
DST	Age	−0.390	−0.514	−4.532	< 0.001	0.857	0.735
	**Left RN**	−0.109	−0.383	−3.375	0.003		
	Education	1.073	0.353	3.250	0.004		
MoCA	Age	−0.179	−0.382	−2.476	0.021	0.724	0.524
	**Left RN**	−0.063	−0.360	−2.338	0.029		
	Education	0.587	0.322	2.148	0.043		
Visuospatial–executive	Age	−0.040	−0.399	−2.883	0.009	0.796	0.633
	Education	0.155	0.400	2.977	0.007		
	**Left RN**	−0.014	−0.375	−2.714	0.013		
Naming	**Right CN**	−0.028	−0.391	−2.243	0.035	0.606	0.367
	**Left RN**	−0.010	−0.382	−2.190	0.039		
Language	**Left SN**	−0.011	−0.491	−2.704	0.013	0.491	0.241

## 4 Discussion

In this study, patients with liver cirrhosis had lower susceptibility values in bilateral CN, TH, and RN compared with controls. The susceptibility values of bilateral RN and left DN were negatively correlated with the DST scores and/or the visuospatial/executive function scores. Additionally, the susceptibility values of the bilateral DN and SN were negatively correlated with the language scores, and the susceptibility values of the right CN were negatively correlated with the naming scores. Our further multivariate analysis suggested the predictive value of the susceptibility values of left RN, right CN, and left SN in association with cognitive disorder in liver cirrhosis.

Previous studies using T2*‐weighted gradient echo imaging (Liu et al. 2013; Lin et al. 2013) and single echo susceptibility mapping (Xia et al. 2015) showed altered phase values and susceptibility values in different brain regions of cirrhotic patients. Although the measured brain regions and distribution patterns of the values were not entirely consistent, they all suggested iron deposition in DGM. However, our results showed an opposite trend with decreased susceptibility values in most selected brain regions, especially in CN, TH, and RN. Different study populations and quantitative MRI techniques may partly explain this difference. The decreased susceptibility value of TH can be explained by the redistribution of iron or cancellation of susceptibilities by diamagnetic myelin. Indeed, decreased susceptibility values in DGM are uncommon in central nervous system diseases, but have also been found in children with autism or attention‐deficit hyperactivity disorder (Tang et al. 2022; Tang et al. 2021), patients with Cushing's disease (Jiang et al. 2022; Jiang et al. 2023), and hemodialysis patients with restless legs syndrome (Wang et al. 2020). Generally, decreased susceptibility values in DGM were attributed to iron deficiency or dysregulation of diamagnetic metals such as calcium (Tang et al. 2022; Tang et al. 2021; Jiang et al. 2022; Jiang et al. 2023; Wang et al. 2020). Notably, there was a large variation (SD) in the susceptibility values of GP and PU in cirrhotic patients. Additionally, we observed a close association between the susceptibility values of GP and PU with both Child–Pugh score and blood ammonia level. Hence, it is postulated that apart from iron deposition, metals such as manganese and calcium may progressively accumulate during the progression of liver cirrhosis. Although elevated blood ammonia levels are commonly associated with hepatic encephalopathy, our findings did not reveal a significant correlation between ammonia levels and neuropsychological scores. This lack of correlation may be attributed to the limited number of patients who underwent ammonia testing and the heterogeneity of underlying causes. Further investigation specifically focusing on hepatic encephalopathy and its relationship with ammonia will provide us with more comprehensive insights into this matter.

A recent study by Lee et al. (2018) found that cirrhotic patients with pallidal T1 hyperintensity had significantly lower susceptibility values in the GP and DN than those without, which provides new interpretive clues for our findings. Pallidal T1 hyperintensity is considered an indicator of manganese accumulation in patients with chronic liver failure (Klos et al. 2006; Krieger et al. 1995). Manganese is also a paramagnetic metal that shares the same cellular transporters with iron, which may account for similar deposition patterns of both in the brain (Chen et al. 2019). Advanced liver dysfunction can disrupt the competitive balance between iron and manganese due to insufficient blood manganese clearance and abnormal iron regulation (Krieger et al. 1995; Nemeth et al. 2004). A postmortem study showed that iron concentration in DGM of patients with liver failure not only did not decrease but even increased (Klos et al. 2006). Therefore, we speculate that the interaction between iron and manganese may be a potential mechanism for the decreased susceptibility values of DGM. In this study, only five patients were visually identified as pallidal T1 hyperintensity, but we suggest that invisible manganese overload may still play a role in the altered regional susceptibility. Further quantitative methods, such as T1 mapping (Zhang et al. 2021), are needed to verify this phenomenon and elucidate how the interaction between manganese and iron accumulation in vivo changes the susceptibility values.

The DGM plays a crucial physiological role in motor control, coordination, balance, and sensory processing. Extensive research on various neurodegenerative diseases has demonstrated that iron deposition–induced magnetic susceptibility alteration in the DGM serves as a biomarker for the pathogenesis of AD and PD, closely associated with cognitive impairment and movement disorders (Uchida, Kan, Sakurai, Oishi et al. 2022; Uchida, Kan, Sakurai, Horimoto et al. 2020, 2022, 2020, 2022, 2019). This study demonstrated that the susceptibility values of the left RN negatively predicted the DST, MoCA, and visuospatial–executive function scores in cirrhotic patients. In addition, increased susceptibility values of the RN and DN were associated with decreased DST and visuospatial–executive scores. RN and DN are subcortical structures separately located in the ventral midbrain and cerebellum and are associated with motor control, coordination, planning, and execution (Dacre et al. 2021; Basile et al. 2020). Via the superior cerebellar peduncle, the DN and RN on one side and the inferior olivary nucleus on the other comprise the so‐called Guillain–Mollaret triangle, which is the preferred nucleus axis involved in hepatic encephalopathy (Kim et al. 2008). Iron and manganese are commonly deposited metals in this region in chronic liver failure, which are cofactors of many enzymes that support normal physiological activities of brain cells and are neurotoxic when accumulated (Klos et al. 2006; Chen et al. 2019; Maeda et al. 1999).

We suggest that the susceptibility values reflecting metal deposition in the RN and DN, mostly RN, may be indicative of cirrhosis‐associated psychomotor and executive dysfunction.

MoCA sub‐domain analysis also showed that the susceptibility values of the right CN and left RN were predictors of naming function, and that of the left SN is even an independent predictor of language function. In addition, the susceptibility values of SN, DN, and CN were negatively correlated with language and naming performance. Although there were no similar QSM studies before, there is much evidence that the CN, RN, SN, and DN are connected with the superior cerebral language center or participate in the generation and processing of language and naming. A study using depth electrodes to record neuronal activity showed that object naming caused an increase in CN firing rates (Abdullaev and Melnichuk 1997). Infarction involving the RN can result in non‐motor symptoms such as decreased verbal fluency (Basile et al. 2020). A DTI study showed a significant relationship between language scores and fractional anisotropy (FA) of left SN (Sengul et al. 2022). For DN, a 7T functional MRI study demonstrated that distinct regions of the cerebellar cortex and DN are associated with motor‐linguistic control and verb generation (Thürling et al. 2011). Another DTI study reconstructed the connection fibers between DN and contralateral frontal cortex including the Broca language area (Ji et al. 2019). Furthermore, a previous study assessed the manganese accumulation in welders by measuring T1 relaxation time and found that lower T1 relaxation time in the CN and SN was associated with poorer performance on tests of verbal fluency, verbal learning, memory, and perseveration (Bowler et al. 2018). Accordingly, we suggest that the susceptibility values of CN, RN, SN, and DN are negatively associated with language and naming abilities in cirrhotic patients.

This study explored the relationship between the susceptibility values quantified by QSM and cognitive function in patients with liver cirrhosis. A previous T2^*^ MRI study showed that the phase values of the left PU and right FWM positively correlated with DST scores and negatively correlated with NCT‐A scores, suggesting that iron deposition in the frontal cortical–basal ganglia circuits might underlie the cognitive decline in MHE patients (Liu et al. 2013). However, our results did not show similar correlations. Quantification methods may be one of the reasons for the inconsistency. QSM resolves the non‐local and orientation dependency nature of phase values and quantifies iron in vivo in DGM more accurately than T2^*^ MRI (Haacke et al. 2015; Langkammer et al. 2012; Wang and Liu 2015). In addition to different quantification methods, the selected patients may also explain the discrepancy in the relationship between neuropsychological tests and MRI measurements. Therefore, further studies comparing QSM and T2^*^ MRI in populations with cirrhosis and MHE are needed.

We acknowledge that this preliminary study has several limitations. First, the relatively small sample size limited our further stratified analysis of different severities or phenotypes of cirrhosis. Second, the heterogeneous etiology may affect the characteristics of brain metal deposition. Large sampled studies of a single etiology can avoid the interference of different metals on susceptibility measurements. Third, a single quantitative method such as QSM may not comprehensively reflect the brain metal deposition in cirrhosis. Other quantitative MRI methods, such as T1 mapping, can help identify and differentiate metal deposits in cirrhosis.

## 5 Conclusion

QSM is a feasible technique for measuring brain metal accumulation in patients with liver cirrhosis. Decreased susceptibility values in the bilateral CN, TH, and RN may be due to interaction between iron and manganese. The susceptibility values of multiple DGM are associated with cognitive impairment in cirrhotic patients. Multiple linear regression analysis suggests that the susceptibility values of the left RN, right CN, and left SN are predictors of psychomotor and executive function, naming, and language function. The susceptibility values of DGM, especially RN, are potential quantitative indicators of cognitive impairment in patients with liver cirrhosis.

## Author Contributions

**Wenjun Wu**: conceptualization, data curation, formal analysis, funding acquisition, investigation, methodology, software, visualization, writing–original draft. **Yu Su**: data curation, investigation, methodology, software, visualization, writing–review and editing. **Ziji Qin**: data curation, methodology, software, visualization, writing–review and editing. **Jiamin Kang**: data curation, methodology, software, writing–review and editing. **Dongqiao Xiang**: methodology, software, writing–review and editing. **Dingxi Liu**: methodology, software, validation, writing–review and editing. **Chuansheng Zheng**: resources, software, supervision, writing–review and editing. **E. Mark Haacke**: conceptualization, resources, software, writing–review and editing. **Lixia Wang**: conceptualization, funding acquisition, investigation, methodology, project administration, resources, supervision, validation, writing–review and editing.

## Ethics Statement

This study was approved by the Institutional Ethics Committee (No. UHCT21810). All subjects were informed and signed written consent before participation.

## Conflicts of Interest

The authors declare no conflicts of interest.

### Peer Review

The peer review history for this article is available at https://publons.com/publon/10.1002/brb3.70240.

## Data Availability

The data that support the findings of this study are available from the corresponding author upon reasonable request.
